# Prospective Observational Study Comparing Systemic Inflammatory Responses Across Different Perfusion Systems During Isolated On-Pump Coronary Artery Bypass Grafting

**DOI:** 10.1093/icvts/ivaf221

**Published:** 2025-09-24

**Authors:** Kaan Kırali, Sibel Aydın, Ayhan Güneş, Mehmet Aksüt, Eray Metin Güler, Mustafa Emre Gürcü

**Affiliations:** Koşuyolu YİEA Hastanesi, Denizer Caddesi No: 2, Cevizli Kavşağı, 34865, Kartal, Istanbul, Türkiye; Koşuyolu YİEA Hastanesi, Denizer Caddesi No: 2, Cevizli Kavşağı, 34865, Kartal, Istanbul, Türkiye; Koşuyolu YİEA Hastanesi, Denizer Caddesi No: 2, Cevizli Kavşağı, 34865, Kartal, Istanbul, Türkiye; Koşuyolu YİEA Hastanesi, Denizer Caddesi No: 2, Cevizli Kavşağı, 34865, Kartal, Istanbul, Türkiye; Koşuyolu YİEA Hastanesi, Denizer Caddesi No: 2, Cevizli Kavşağı, 34865, Kartal, Istanbul, Türkiye; Koşuyolu YİEA Hastanesi, Denizer Caddesi No: 2, Cevizli Kavşağı, 34865, Kartal, Istanbul, Türkiye

**Keywords:** systemic inflammatory response, cardiopulmonary bypass, minimal invasive extracorporeal circulation, hybrid system, coronary artery bypass grafting

## Abstract

**Objectives:**

Minimal invasive Extracorporeal Circulation (MiECC) and Hybrid System (HS) have been introduced to potentially reduce the inflammatory response compared to conventional Cardiopulmonary Bypass (cCPB). The HS combines elements of conventional and minimized circuits, including a collapsible reservoir, integrated arterial filter, and hypobaric oxygenator, allowing rapid conversion and air embolism control. This study aims to provide a comparative analysis of biomarkers of systemic inflammatory response induced by MiECC, HS, and cCPB systems in isolated coronary artery bypass grafting (CABG) patients.

**Methods:**

This prospective observational study included a total of 66 patients who underwent isolated on-pump CABG performed under aortic cross-clamp. Systemic inflammatory markers (interleukin [IL]-1β, IL-6, IL-8, tumour necrosis factor [TNF]-α, lactate enzyme [LE], and hypoxia inducible factor [HIF]-1α) and oxidative status were measured at 5 intervals: preoperative (pre-pump), intraoperative (on-pump), and at 6, 12, and 24 h postoperatively (post-pump).

**Results:**

A total of 66 patients were enrolled: MiECC (n = 20), HS (n = 22), and cCPB (n = 24). Notably, 2 patients initially assigned to MiECC required intraoperative conversion to cCPB due to haemodynamic instability. Both MiECC and HS groups consistently showed lower levels of systemic inflammatory biomarkers and oxidative stress indicators at all intraoperative and postoperative time points compared to cCPB. For instance, IL-6 levels at 6 h post-op were 292 pg/mL in MiECC, 311 pg/mL in HS, and 514 pg/mL in cCPB; oxidative stress index values at the same time point were 70 in MiECC, 66 in HS, and 142 in cCPB. Haemoglobin decline was least pronounced in the MiECC group, and red blood cell transfusion was required in 50% of cCPB patients, compared to 10% in MiECC and 13.6% in HS. HIF-1α levels were higher in HS than MiECC at 12 h post-op (3.8 vs 2.6 ng/mL). No substantial differences were observed between groups in troponin, creatinine, or lactate values.

**Conclusions:**

MiECC and HS show a similar profile in alleviating systemic inflammation, with notable reductions in inflammatory biomarkers and key clinical oxidative outcomes compared with cCPB. These results underscore the potential of MiECC and HS to improve clinical recovery by minimizing the inflammatory effect in on-pump CABG procedures.

## Graphical abstract

**Figure:**
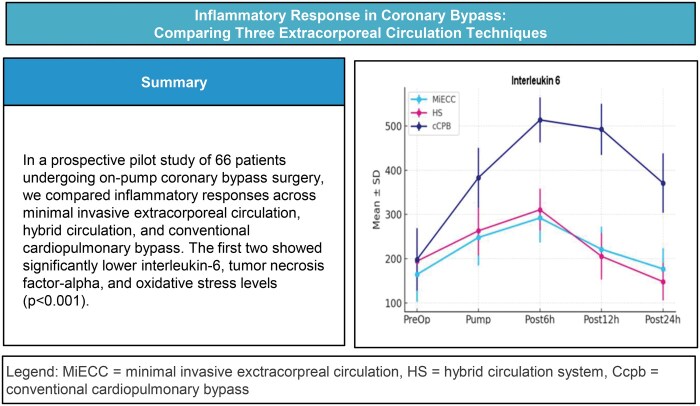

## INTRODUCTION

Extracorporeal circulation (ECC) is essential in on-pump cardiac surgery, as it enables surgeons to maintain whole body tissue perfusion and oxygenation via haemodynamic stabilization while the heart is temporarily stopped. However, ECC is associated with the induction of a substantial systemic inflammatory response due to the exposure of the blood to non-physiological surfaces, blood-air interaction, ischaemia-reperfusion injury, and other procedural factors. This systemic inflammatory response can escalate into a more severe condition known as Systemic Inflammatory Response Syndrome (SIRS), which is characterized by widespread inflammation affecting multiple organs, leading multiorgan dysfunction, prolonged recovery times, ultimately impacting patient outcomes and increasing healthcare costs.^1^ The initial reason for developing new perfusion systems was to inactivate the contact coagulation pathway activated by the interaction between blood and non-endothelial surfaces using biocompatible products.^2^^,^^3^ The Hybrid System (HS) aims to optimize efficiency and prevent inflammation by combining Minimal invasive Extracorporeal Circulation (MiECC) and cCPB elements, while minimizing the risk of gas embolism with the hypobaric oxygenator in the circuit.^4^^,^^5^ This study aimed to investigate differences in systemic inflammatory responses among 3 ECC configurations MiECC, HS, and cCPB in patients undergoing isolated on-pump coronary artery bypass grafting (CABG). We hypothesized that the HS would elicit a comparable inflammatory response to MiECC, and a markedly lower response than cCPB.

## METHODS

### Study design

This single-centre prospective observational study that included 66 consecutive patients undergoing elective isolated CABG surgery via ECC, namely with 3 different techniques was conducted at Koşuyolu High Specialization Education and Research Hospital, Istanbul, Türkiye between February 2024 and August 2024.

### Ethics statement

The study was approved by the Institutional Review Board of Koşuyolu High Specialization Education and Research Hospital (Approval No. 814-092024). Written informed consent was obtained from all participants prior to enrolment. The study adhered to the ethical principles outlined in the Declaration of Helsinki. In addition, any data and biological materials collected from participants were stored in accordance with institutional guidelines and ethical standards. While no formal biobank was established, all serum samples used for biomarker analysis were stored at −80°C for the purpose of this specific study and were not intended for indefinite or future use. The storage and use of these samples were reviewed and approved by the same ethics committee in line with the WMA Declaration of Taipei. No reuse of data or biological material beyond the current study is planned without further ethical approval.

We implemented a structured sequential allocation process to ensure balanced distribution of participants across groups. Specifically, patients were assigned to 1 of the 3 ECC systems (MiECC, HS, or cCPB) in a fixed rotating order based on the sequence of enrolment: the first patient was allocated to MiECC, the second to HS, the third to cCPB, and the cycle repeated accordingly. This approach allowed for uniform group sizes while minimizing selection bias. As a prospective observational study, a formal sample size calculation was not performed; the chosen number of 66 patients was considered adequate to explore inflammatory trends across perfusion systems. The allocation of participants to each group was implemented a sequential rotation to provide a systematic and balanced allocation of participants across all 3 groups, minimizing potential selection bias and ensuring a uniform distribution over time: the first patient was assigned to the MiECC group, the second to the HS group, and the third to the cCPB group. After the third patient, the cycle repeated in the same order.

### ECC techniques

#### The MiECC system (LivaNova, Sorin Inspire)

The setup used in this study is similar Type I, with only exception being a venous bubble trap/air removing device (designated by us as Type Ib) and it can be easily converted to cCPB circuit.^6^ The MiECC System features a closed-loop circuit designed with a Sorin Inspire 6 F oxygenator integrated with an arterial filter, a centrifugal pump, and physio-coated tubing to minimize blood and foreign surface interaction, potentially reducing the systemic inflammatory response.

#### The Hybrid System (Spectrum Medical)

This system combines features of both MiECC and cCPB systems to offer adaptability to complex cases. It incorporates a dual-chamber venous reservoir with a collapsible soft bag, a VT200 oxygenator integrated with an arterial filter, a roller pump, and physio-coated tubing. The system facilitates the management of vented and suctioned blood separately from the venous return, but by collecting them in the soft bag, it provides flexible volume control and enables substantial venting of air in the venous soft bag reservoir to minimize blood and air interaction. It is compatible with vacuum assist venous drainage approaches and allows smooth transitions from a mini bypass to full drainage as needed, making it adaptable to various intraoperative demands while aiming to minimize haemodynamic instability.^7^ In addition, hypobaric and novel dual-chamber oxygenator design with air-trap prevents micro air bubble embolism.

#### cCPB system (LivaNova, Sorin Inspire)

The configuration utilizes a hard-shell venous reservoir with a vacuum assist venous drainage approach,^8^ a Sorin Inspire 6 F oxygenator integrated with an arterial filter, a centrifugal pump, and physio-coated tubing. This setup accumulates venous return along with all vented and suctioned blood, providing the most effective circulatory support when acute volume replacement is required, but the large surface area leading to blood and air contact results in increased air exposure to blood. The retrograde autologous priming was not applied in any group. Circuits configurations comply with European Board of Cardiovascular Perfusion (EBCP) and MiECTiS (Minimal Invasive Extracorporeal Technologies international Society) guidelines.

### Surgical technique

All CABG procedures were performed by the same team to ensure consistency. After the standard median sternotomy and systemic heparinization under the general anaesthesia, all arterial and venous grafts were harvested and ECC was initiated using an aortic cannula (20 or 22 Fr) inserted into the ascending aorta for systemic arterial flow, and a dual-stage venous cannula (32/40 Fr) through the right atrial appendage for systemic venous return. During ECC, heparin was administered to maintain that the activated clotting time (ACT) for cCPB and HS was > 480 s, but for the MiECC system, the ACT was maintained below 300 s.^9^ After aortic cross clamping, Del Nido cardioplegia solution (1250 ml; crystalloid-blood ratio 4:1; < 10°C) was administered through an antegrade cardioplegia cannula placed just above the aortic root to achieve and maintain myocardial arrest.

### Anaesthesia management

Preoperative assessment included optimization of comorbidities and premedication with midazolam (1-2 mg IV) if needed. Standard monitoring methods (eg, electrocardiography, pulse oximetry, arterial pressure, and central venous pressure) were used, with bispectral index (BIS) for depth of anaesthesia and near infrared spectroscopy (NIRS) for cerebral oxygenation. Induction medications included midazolam (0.03-0.05 mg/kg IV), fentanyl (5-10 µg/kg IV), and either propofol (0.5-1 mg/kg IV) or etomidate (0.2-0.3 mg/kg IV) based on patient stability, followed by rocuronium (0.6-1 mg/kg IV) for intubation. Anaesthesia maintenance was provided by desflurane (0.5-1.0 MAC) inhalation or by intravenous anaesthesia only along with continuous of remifentanil (1-5 µg/kg/h) and rocuronium infusions accompanied by BIS and NIRS monitoring,

### Data collection

Preoperative patient demographics (age, gender, body mass, left ventricular function), intraoperative variables (bypass number, ECC and ACC times), and postoperative clinical outcomes (drainage, blood transfusion, mechanical ventilation, ICU and hospital stay durations) were measured to compare clinical effects of ECC systems. Intraoperative priming volume and partial arterial pressure of oxygen (PaO_2_) were also measured to compare secondary clinical effect on inflammation. Blood samples were collected from patients to measure haemoglobin and platelet values, biochemical variables (lactate, creatinine, troponin, c-reactive protein, and lactate dehydrogenase), and various inflammatory biomarkers at 5 different time periods. The samples were collected at baseline (pre-pump), during ECC (20 min after initiation), and postoperatively at 6 h, 12 h, and 24 h. The samples were placed in biochemistry tubes containing a gel clot activator. After centrifuging the samples at 3000 × g for 10 min, the serums were separated and stored at ‐80°C until biochemical analyses were performed.

### Enzyme-Linked Immunosorbent Assay

Levels of interleukin (IL)-1β (E0143Hu, BT Lab, China), IL-6 (E0090Hu, BT Lab, China), IL-8 (E0089Hu, BT Lab, China), tumour necrosis factor-α (TNF-α; E0082Hu, BT Lab, China), leucocyte elastase (HLE; MBS729215, My BioSource, USA), and hypoxia-inducible factor-1 alpha (HIF-1α; E0422Hu, BT Lab, China) in serum samples were determined using enzyme-linked immunosorbent assay (ELISA) kits according to the manufacturer’s instructions. For each assay, 40 μL of serum sample and 10 μL of a parameter-specific antibody were pipetted into each well of a microplate.

### Oxidative stress analysis

To assess the balance between oxidative and antioxidative activity, this study measured the total oxidant status (TOS) and total antioxidant status (TAS) levels in serum. These measurements were conducted using photometric methods with commercially available assay kits from Rel Assay Diagnostics. TOS levels were expressed in micromoles of hydrogen peroxide equivalents per litre (µmol H_2_O_2_ equivalent/l). TAS levels, reflecting the serum’s antioxidant capacity, were expressed in millimoles of ascorbic acid equivalents per litre (mmol ascorbic acid equivalent/l). The oxidative stress index (OSI) was calculated as the ratio of TOS to TAS and expressed in arbitrary units (AU).

### Statistical analysis

Statistical analysis was conducted using SPSS software (version 28). The distribution of data was evaluated for normality using the Shapiro-Wilk test, and homogeneity of variances was assessed using the Levee’s test. Continuous variables were presented as mean ± standard deviation, and categorical variables are presented as frequency (percentage). For inter-group comparisons of continuous variables, One-way analysis of variance (ANOVA) was employed. In cases where ANOVA indicated statistically significant differences, Tukey’s honestly significant difference (HSD) post-hoc test was applied to determine the specific group differences. For categorical variables, the Chi-square test or Fisher’s exact test was used as appropriate based on expected cell counts. A simplified repeated-measures ANOVA was applied to compare changes in inflammatory and oxidative markers over time within and between groups. The threshold for statistical significance was set at *P* < .05 for all analyses.

## RESULTS

### Patient demographics, surgical characteristics, and clinical outcomes

Demographic and baseline preoperative characteristics of patients in all 3 groups were similar; the average age of the patients was 60 years and the majority of them was male, but the cCPB group had significantly higher female patients (*P* = .045) (**Table 1**). Given the exploratory nature of this study, no multivariate models were constructed. However, subgroup trends by age and sex were checked descriptively and no major imbalance was observed. The cCPB system had statistically the worst value compared to MiECC and HS systems, that is, higher PaO_2_ (*P* = .001) value that could aggravate SIRS (**Table 2**). Although there was no difference between the groups in terms of mean ACC and ECC times, these times were shorter in the HS group, which required less multivessel bypass. The mean mechanical ventilation time, length of stay in ICU, and discharge time were similar in all groups (**Table 3**). Considering the haematological values, although all 3 systems caused mild reduction in platelet count, this rate was less in the MiECC group, <10% than the others, but there was no statistical difference. Postoperative recovery in platelet counts was sufficient in all 3 groups without any statistically difference (**Table 4**).

**Table 1.** Demographic Characteristics of Patients in Each Group

Preoperative characteristic	MiECC	HS	cCPB	*P*
	(n = 20)	(n = 22)	(n = 24)	
Mean age (years)	59.2 ± 8.1	63.8 ± 6.8	61.4 ± 9.8	.273
Male sex	17 (85%)	19 (86.3%)	14 (58.3%)	**.045**
Mean BSA (m^2^)	1.94 ± 0.13	1.94 ± 0.13	1.91 ± 0.14	.640
Mean LVEF (%)	57.6 ± 10.1	59.5 ± 11.6	57.0 ± 13.4	.765
Hypertension	9 (45%)	10 (45.4%)	11 (45.8%)	.996
Diabetes mellitus	6 (30%)	7 (31.8%)	8 (33.3%)	.958

Abbreviations: BSA, body surface area; cCPB, conventional Cardiopulmonary Bypass, HS, Hybrid System; LVEF, left ventricular ejection fraction; MiECC, minimally invasive extracorporeal circulation.

**Table 2.** ECC System Variables

	MiECC	HS	cCPB	One-way ANOVA	Post-hoc comparison test (Tukey)		
	(n = 20)	(n = 22)	(n = 24)				
Variable				*P*	MiECC vs HS	MiECC vs cCPB	HS vs cCPB
PaO_2_ (mmHg)	210.9 ± 49.6	178.9 ± 68.8	270.8 ± 48.2	**.001**	0.217	**0.002**	**<0.001**
PV (mL)	1592 ± 474	1957 ± 515	2115 ± 629	**.011**	0.98	**0.009**	0.608

Abbreviations: cCPB, conventional Cardiopulmonary Bypass; HS, Hybrid System; MiECC, minimally invasive extracorporeal circulation; PaO_2_, partial arterial oxygen pressure; PV, priming volume.

**Table 3.** Postoperative Processes

	MiECC	HS	cCPB	*P*
	(n = 20)	(n = 22)	(n = 24)	
Postoperative drainage (mL)	573 ± 495	588 ± 337	643 ± 289	.815
Mechanical ventilation (h)	12.5 ± 2.4	11.9 ± 3.7	12.4 ± 3.8	.828
Length of stay in ICU (days)	1.9 ± 1.2	1.7 ± 1.2	2.2 ± 1.8	.500
Hospital stay (days)	6.5 ± 2.1	6.4 ± 1.9	6.1 ± 1.5	.729

Abbreviations: cCPB, conventional Cardiopulmonary Bypass; HS, Hybrid System; ICU, intensive care unit; MiECC, minimally invasive extracorporeal circulation.

**Table 4.** Haematological Variables Obtained at Specified Time Periods

	MiECC (n = 20)	HS (n = 22)	cCPB (n = 24)	One-way ANOVA	Post-hoc comparison tests (Tukey)		
	(n = 20)	(n = 22)	(n = 24)				
				*P*	MiECC vs HS	MiECC vs cCPB	HS vs cCPB
***Haemoglobin (g/dl)***							
Before ECC	11.99 ± 1.59	12.14 ± 1.95	11.79 ± 1.47	.726	–	**-**	**-**
During ECC	9.88 ± 1.60	8.62 ± 1.28	8.82 ± 1.28	**.007**	**0.01**	**0.028**	0.887
Postoperative 6 h	10.76 ± 1.38	10.29 ± 1.14	9.46 ± 1.58	**.008**	0.44	**0.006**	0.123
Postoperative 12 h	10.03 ± 1.19	10.01 ± 0.95	9.41 ± 1.52	.118	–	**-**	**-**
Postoperative 24 h	9.03 ± 1.13	9.10 ± 0.85	8.66 ± 1.08	.341	–	**-**	**-**
***Platelets (×10^9^/l)***							
Before ECC	222.8 ± 75.1	207.4 ± 49.5	192.0 ± 53.2	.249	–	**-**	**-**
During ECC	199.9 ± 63.6	162.4 ± 57.4	164.3 ± 66.9	.105	–	**-**	**-**
Postoperative 6 h	233.1 ± 70.6	215.5 ± 73.8	189.7 ± 56.9	.139	–	**-**	**-**
Postoperative 12 h	224.1 ± 65.1	211.4 ± 64.3	188.7 ± 70.4	.204	–	**-**	**-**
Postoperative 24 h	216.0 ± 67.2	194.8 ± 59.9	193.7 ± 70.9	.509	–	**-**	**-**
***RBC transfusion (patient number)***	2/20 (10%)	3/22 (13.6%)	12/24 (50%)	**.003**			
***Platelets transfusion (patient number)***	0	0	0	**-**			

Abbreviations: cCPB, conventional Cardiopulmonary Bypass; HS, Hybrid System; ICU, intensive care unit; MiECC, minimally invasive extracorporeal circulation.

**Figure 1.**
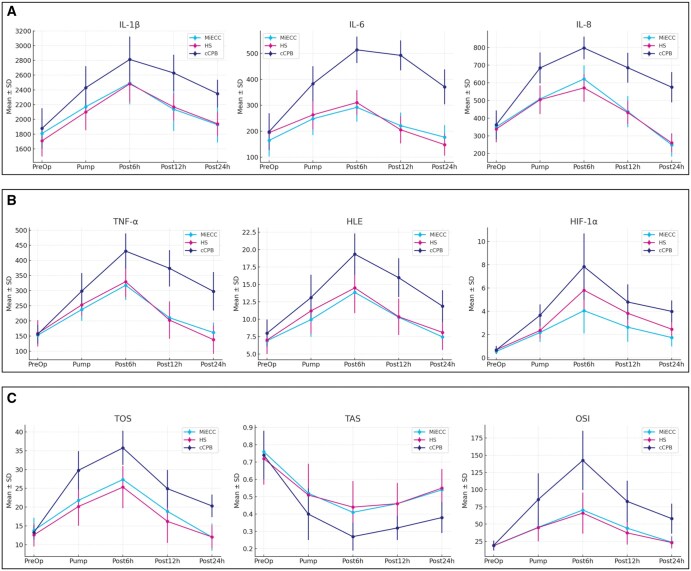
Peri-, Intra-, and Postoperative Trends. (**A**) Systemic inflammatory cytokines, (**B)** cellular and hypoxia markers; and **(C)** oxydative stress and antioxidants in patients undergoing isolated on-pump CABG with different extracorporeal circulation (ECC) systems. Abbreviations: cCPB, conventional cardiopulmonary bypass; HS, hybrid system; MiECC, minimal invasive extracorporeal circulation

More patients in the cCPB group required red blood cell (RBC) transfusion than in the other 2 groups (**Figure 1**). The study employed a Chi-square analysis to evaluate the distribution of RBC units usage across different extracorporeal circuit types. The results revealed a statistically significant association between circuit type and RBC usage (χ^2^ = 11.663, df = 2, *P* = .003). Subgroup analysis showed no statistically significant difference in RBC usage between the MiECC (10.0%) and HS (13.6%) configurations (*P* = 1.00). However, the cCPB demonstrated a markedly higher RBC usage rate (50.0%) compared to both MiECC (*P* = .005) and HS (*P* = .009). Platelet transfusion was not required in any group.

### Biochemical measurements over time periods

In the biochemical analysis performed to evaluate the effect of all 3 systems on general body perfusion, troponin levels indicating heart damage, creatinine indicating kidney damage, and lactate levels indicating tissue perfusion deficiency gave similar results in all groups at all measurement times, without indicating a negative effect (**Table 5**). Additionally, no significant difference was found in favour of any of them.

### Oxidative stress measurements

When oxidative stress biomarkers (eg, TOS, TAS, and OSI) were evaluated, a statistically significant negative effect of ECC was observed in almost all of the measurements in the cCPB group compared to the MiECC and HS groups (**Table 6**). This means that TOS was significantly higher (*P* < .001) in the cCPB group compared to MiECC and HS groups at each time period after ECC initialization. Activated antioxidant response state to prevent this, TAS was significantly decreased in the cCPB group than the other groups (*P* < .001). OSI, which is the net result of the conflict of these 2 statuses, was higher in the cCPB group, and both MiECC and HS groups showed a moderated oxidative response, with lower OSI levels at each time point postoperatively. All oxidative (TOS, OSI) and antioxidative (TAS) markers showed significant changes after the onset of ECC. Oxidative markers increased and peaked at 6 h postoperatively, while TAS decreased during the same period, then gradually normalized by 24 h. At the 24th postoperative hour, while *all biomarkers* still remained conspicuously elevated/depressed within the cCPB group, the values in the MiECC and HS groups came very close to the baseline.

**Table 5.** Biochemical Parameters Obtained at Specified Time Periods

	MiECC	HS	cCPB	One-way ANOVA*P*	Post-hoc comparison tests (Tukey)		
	(n = 20)	(n = 22)	(n = 24)		MiECC vs HS	MiECC vs cCPB	HS vs cCPB
Lactate (before ECC)	1.68 ± 0.60	1.57 ± 0.54	1.87 ± 0.83	.320	-	**-**	**-**
Lactate (during ECC)	2.11 ± 0.85	2.10 ± 0.98	2.21 ± 1.43	.950	-	**-**	**-**
Lactate (postop. 6 h)	4.04 ± 1.40	2.98 ± 1.30	3.93 ± 1.90	.059	–	**-**	**-**
Lactate (postop. 12 h)	3.51 ± 1.76	3.07 ± 1.50	3.35 ± 1.83	.627	–	**-**	**-**
Lactate (postop. 24 h)	2.50 ± 0.93	2.01 ± 0.70	2.64 ± 1.33	.104	–	**-**	**-**
Creatinine (before ECC) (mg/dl)	0.79 ± 0.16	0.91 ± 0.29	1.02 ± 0.54	.156	–	**-**	**-**
Creatinine (during ECC)	0.70 ± 0.16	0.76 ± 0.25	0.81 ± 0.43	.490	–	**-**	**-**
Creatinine (postop. 6 h	0.84 ± 0.21	1.00 ± 0.33	1.03 ± 0.46	.210	–	**-**	**-**
Creatinine (postop. 12 h)	0.89 ± 0.18	1.02 ± 0.42	1.12 ± 0.52	.199	–	**-**	**-**
Creatinine (postop. 24 h)	0.79 ± 0.16	1.04 ± 0.58	1.18 ± 0.58	.051	–	**-**	**-**
Troponin (before ECC) (ng/ml)	0.01 ± 0.01	0.02 ± 0.03	0.05 ± 0.17	.446	–	**-**	**-**
Troponin (during ECC)	0.92 ± 0.91	1.11 ± 2.28	1.35 ± 2.18	.735	–	**-**	**-**
Troponin (postop. 6 h)	4.64 ± 3.42	3.73 ± 3.55	3.28 ± 3.16	.524	–	**-**	**-**
Troponin (postop. 12 h)	5.35 ± 6.15	4.21 ± 4.33	5.04 ± 5.68	.818	–	**-**	**-**
Troponin (postop. 24 h)	4.1 ± 4.82	3.66 ± 4.97	3.98 ± 4.31	.970	–	**-**	**-**
CRP (before ECC) (mg/l)	6.50 ± 6.30	10.31 ± 14.42	12.02 ± 21.52	.526	–	**-**	**-**
CRP (during ECC)	4.84 ± 4.38	6.55 ± 9.46	22.91 ± 69.35	.302	–	**-**	**-**
CRP (postop. 6 h)	22.30 ± 15.44	16.19 ± 17.94	22.59 ± 27.43	.533	–	**-**	**-**
CRP (postop. 12 h)	70.95 ± 25.34	66.41 ± 24.69	66.96 ± 32.55	.753	–	**-**	**-**
CRP (postop. 24 h)	170.43 ± 45.97	160.70 ± 35.05	175.52 ± 40.49	.456	–	**-**	**-**
LDH (before CPB) (IU/l)	143.85 ± 31.82	141.95 ± 22.93	142.17 ± 26.92	.929	–	**-**	**-**
LDH (during ECC)	156.45 ± 25.54	269.64 ± 110.80	329.52 ± 108.51	**<.001**	**<0.001**	**<0.001**	**<0.001**
LDH (postop. 6 h)	287.20 ± 81.02	414.05 ± 177.20	425.74 ± 116.52	**.002**	**0.010**	**0.004**	0.954
LDH (postop. 12 h)	263.70 ± 72.86	359.43 ± 88.26	437.17 ± 211.21	**.001**	0.105	**<0.001**	0.180
LDH (postop. 24 h)	254.25 ± 74.55	317.19 ± 109.71	570.52 ± 1015.31	.214	–	**-**	**-**

Abbreviations: cCPB, conventional Cardiopulmonary Bypass; CRP, C-reactive protein; HS, Hybrid System; LDH, lactate dehydrogenase; MiECC, minimally invasive extracorporeal circulation.

**Table 6.** Inflammatory Biomarkers Obtained at Specified Time Periods

Biomarker	Time period	MiECC	HS	cCPB	*P*	Post-hoc comparison tests (Tukey)		
		(n = 20)	(n = 22)	(n = 24)		MiECC vs HS	MiECC vs cCPB	HS vs cCPB
IL-1β (pg/mL)	Before ECC	1809.23 ± 224.05	1708.7 ± 209.63	1877.21 ± 274.66	.079	–	–	–
	During ECC	2172.56 ± 257.04	2101.12 ± 249.21	2430.23 ± 291.63	**<.001**	0.677	**0.009**	**<0.001**
	Postop. 6 h	2492.47 ± 287.46	2481.75 ± 249.88	2812.73 ± 308.32	**<.001**	0.992	**0.002**	**0.001**
	Postop. 12 h	2137.49 ± 293.79	2169.28 ± 186.85	2629.82 ± 246.48	**<.001**	0.916	**<0.001**	**<0.001**
	Postop 24 h	1928.23 ± 242.41	1940.69 ± 166.09	2349.45 ± 187.69	**<.001**	0.981	**<0.001**	**<0.001**
IL-6 (pg/mL)	Before ECC	164.57 ± 61.17	194.51 ± 65.29	198.34 ± 70.65	.222	–	–	–
	During ECC	248.12 ± 63.16	263.27 ± 56.37	383.26 ± 67.59	**<.001**	0.726	**<0.001**	**<0.001**
	Postop. 6 h	292.07 ± 54.65	310.64 ± 47.53	513.51 ± 50.57	**<.001**	0.486	**<0.001**	**<0.001**
	Postop. 12 h	221.39 ± 50.93	205.55 ± 52.52	492.26 ± 57.57	**<.001**	0.641	**<0.001**	**<0.001**
	Postop. 24 h	177.1 ± 46.55	148.33 ± 42.51	370.95 ± 67.24	**<.001**	0.259	**<0.001**	**<0.001**
IL-8 (pg/mL)	Before ECC	349.8 ± 78.46	338.22 ± 74.6	362.03 ± 81.42	.617	–	–	–
	During ECC	508.74 ± 73.11	504.5 ± 80.9	684.35 ± 87.73	**<.001**	0.985	**<0.001**	**<0.001**
	Postop. 6 h	621.85 ± 76.4	571.45 ± 78.43	797.35 ± 63.57	**<.001**	0.083	**<0.001**	**<0.001**
	Postop. 12 h	436.75 ± 87.89	431.79 ± 65.94	685.4 ± 84.15	**<.001**	0.98	**<0.001**	**<0.001**
	Postop. 24 h	249 ± 65.04	259.71 ± 52.23	575.2 ± 85.51	**<.001**	0.891	**<0.001**	**<0.001**
TNF-α (pg/mL)	Before ECC	153.1 ± 30.68	159.14 ± 43.43	156.52 ± 31.32	.868	–	–	–
	During ECC	237.62 ± 36.81	253.25 ± 36.81	298.66 ± 59.97	**<.001**	0.54	**<0.001**	**0.006**
	Postop. 6 h	317.86 ± 48.11	329.86 ± 51.3	430.92 ± 57.89	**<.001**	0.754	**<0.001**	**<0.001**
	Postop. 12 h	210.52 ± 49.14	202.84 ± 61.56	373.94 ± 60.13	**<.001**	0.911	**<0.001**	**<0.001**
	Postop. 24 h	161.67 ± 32.98	138.29 ± 46.74	297.89 ± 63.55	**<.001**	0.361	**<0.001**	**<0.001**
HLE	Before ECC	6.9 ± 1.41	7 ± 1.97	7.99 ± 1.95	.109	–	–	–
	During ECC	9.93 ± 2.46	11.19 ± 3.3	13.1 ± 3.3	**.006**	0.405	**0.005**	0.11
	Postop. 6 h	13.85 ± 2.36	14.5 ± 3.63	19.35 ± 2.96	**<.001**	0.783	**<0.001**	**<0.001**
	Postop. 12 h	10.28 ± 1.8	10.35 ± 2.6	15.97 ± 2.78	**<.001**	0.996	**<0.001**	**<0.001**
	Postop. 24 h	7.45 ± 1.44	8.1 ± 2.51	11.87 ± 2.29	**<.001**	0.649	**<0.001**	**<0.001**
HIF-1α	Before ECC	0.55 ± 0.18	0.69 ± 0.34	0.67 ± 0.32	.313	–	–	–
	During ECC	2.18 ± 0.81	2.35 ± 0.7	3.66 ± 0.92	**<.001**	0.795	**<0.001**	**<0.001**
	Postop. 6 h	4.05 ± 1.96	5.81 ± 1.9	7.83 ± 2.84	**<.001**	**0.047**	**<0.001**	**0.016**
	Postop. 12 h	2.63 ± 1.25	3.82 ± 1.35	4.79 ± 1.51	**<.001**	**0.029**	**<0.001**	0.067
	Postop. 24 h	1.74 ± 0.76	2.46 ± 0.82	3.99 ± 0.93	**<.001**	**0.034**	**<0.001**	**<0.001**
Total oxidant status	Before ECC	13.82 ± 3.36	12.6 ± 3.04	13.27 ± 1.95	.397	–	–	–
	During ECC	21.8 ± 4.19	20.16 ± 5.11	29.79 ± 5.11	**<.001**	0.535	**<0.001**	**<0.001**
	Postop. 6 h	27.35 ± 3.22	25.32 ± 5.62	35.76 ± 4.52	**<.001**	0.348	**<0.001**	**<0.001**
	Postop. 12 h	18.87 ± 4.23	16.2 ± 5.69	24.91 ± 4.99	**<.001**	0.24	**0.001**	**<0.001**
	Postop. 24 h	12.03 ± 3.56	12.07 ± 3.02	20.32 ± 2.98	**<.001**	0.999	**<0.001**	**<0.001**
Total antioxidant status	Before ECC	0.76 ± 0.12	0.72 ± 0.15	0.74 ± 0.14	.652	–	–	–
	During ECC	0.52 ± 0.13	0.51 ± 0.18	0.4 ± 0.15	**.036**	0.994	**0.044**	**0.041**
	Postop. 6 h	0.41 ± 0.09	0.44 ± 0.15	0.27 ± 0.08	**<.001**	0.761	**<0.001**	**<0.001**
	Postop. 12 h	0.46 ± 0.11	0.46 ± 0.12	0.32 ± 0.07	**<.001**	1	**<0.001**	**<0.001**
	Postop. 24 h	0.54 ± 0.12	0.55 ± 0.11	0.38 ± 0.09	**<.001**	0.998	**<0.001**	**<0.001**
Oxidative stress index	Before ECC	18.81 ± 6.08	18.51 ± 6.89	19.02 ± 7.06	.971	–	–	–
	During ECC	45.32 ± 16.78	44.89 ± 20.03	85.74 ± 37.93	**<.001**	0.999	**<0.001**	**<0.001**
	Postop. 6 h	70.39 ± 21.38	65.91 ± 29.79	142.46 ± 42.88	**<.001**	0.903	**<0.001**	**<0.001**
	Postop. 12 h	43.98 ± 17.34	37.15 ± 16.91	82.87 ± 30.14	**<.001**	0.628	**<0.001**	**<0.001**
	Postop. 24 h	23.6 ± 9.2	23.09 ± 7.6	57.9 ± 21.72	**<.001**	0.994	**<0.001**	**<0.001**

Abbreviations: cCPB, conventional Cardiopulmonary Bypass; HIF, hypoxia inducible factor; IL, interleukin; HLE, leucocyte elastase; HS, Hybrid System; MiECC, minimally invasive extracorporeal circulation; TNF, tumour necrosis factor.

## DISCUSSION

This study demonstrates that both ECC techniques, MiECC and HS, offer significant advantages over cCPB in reducing systemic inflammatory responses and oxidative stress activation, which are similar just before ECC, during the first 24 h after ECC initialization. Our findings contribute to the growing body of evidence showing that modifications to extracorporeal technology (eg, short tubing, reduced prime volume, biocompatible circuit surfaces, centrifugal pump, decreased blood-air interaction surface in soft venous reservoir bag, closed circuit design, hypobaric oxygenator) can alleviate the inflammatory burden typically associated with the cCPB system.^10^

HIF‑1α is an oxygen‑sensitive transcription factor that mediates adaptive metabolic responses to hypoxia. An increasing number of studies have demonstrated that HIF‑1α serves a key role in oxygen homeostasis, preventing ischaemia-reperfusion injury, which develops following the recovery of blood flow.^11–15^ Among the biomarkers assessed, pro-inflammatory cytokines (IL-1β, IL-6, IL-8, TNF-α) and oxidative stress indices (TOS, TAS, OSI) showed consistently lower levels in the MiECC and HS groups compared to cCPB, particularly during and after ECC. For instance, IL-6 and TNF-α remained substantially elevated in the cCPB group at 12 and 24 h, indicating prolonged systemic inflammation. Similarly, the oxidative stress index peaked sharply in the cCPB group and remained elevated beyond the first postoperative day, whereas MiECC and HS demonstrated a faster normalization trend.

Although major clinical end-points were not significantly different, the reduction in inflammatory and oxidative markers in MiECC and HS may contribute to faster recovery, lower risk of SIRS, and reduced transfusion needs. Larger trials are needed to confirm these associations with long-term outcomes. Notably, ICU staff were not blinded to group allocation due to documentation of perfusion techniques in operative records. However, transfusion decisions were made according to standard institutional thresholds and protocols, minimizing the potential for subjective bias. Interestingly, the haemoglobin level showed the lowest decrease in the MiECC group rather than the HS and cCPB groups (*P* < .01), while the decrease in the HS and cCPB groups was more pronounced and similar, but insignificant between both these groups (*P* > .05). More patients received RBC transfusion in the postoperative period in the cCPB group rather than the MiECC and HS groups (*P* < .01). The increased need for RBC transfusions in the conventional Cardiopulmonary Bypass (cCPB) group, despite a similar haemoglobin drop, may be probably explained by the stronger inflammatory response observed. This could have promoted systemic vasodilation and increased capillary permeability, leading to intravascular volume depletion and the need for additional fluid replacement.^16^ As a result, haemodilution may have triggered transfusion thresholds earlier in this group. While minimally invasive extracorporeal circulation has a more established evidence base after more than a decade of clinical use,^3^^,^^6^ its limited flexibility in managing significant bleeding or gas embolism within the closed circuit can compromise reproducibility across centres. The HS, although relatively newer, is gaining scientific support and offers greater procedural adaptability, making it a more reproducible and potentially safer option in routine practice.

### Study limitations

The sample size may limit the statistical power for detecting subtle differences in certain outcomes. Two patients in the MiECC group were converted to the cCPB system due to haemodynamic instability, which we could not correct with various manoeuvres, and they were added in cCPB group. Furthermore, while we focused on commonly used inflammatory and oxidative stress markers, additional biomarkers and functional assessments could provide a more comprehensive picture of the systemic response to ECC.

## CONCLUSIONS

The findings of this study have important clinical implications. First, this study highlights the potential benefits of the MiECC and HS compared to cCPB in patients undergoing isolated on-pump CABG. The reduced inflammatory response and oxidative stress seen with MiECC and HS suggest that these technologies will be beneficial for patients undergoing on-pump cardiac surgeries, particularly those at higher risk because of complications related to systemic inflammation and oxidative stress. Second, MiECC and HS strategies prevent the adverse inflammatory response by significantly reducing heterologous blood product transfusion need. Further multicentre studies with larger samples are recommended to validate these findings and to assess clinical outcomes of each system in broader patient populations.

## Data Availability

The data presented in this study are available on request from the corresponding author.
